# The role of the RhoA/Rho kinase pathway in angiogenesis and its potential value in prostate cancer (Review)

**DOI:** 10.3892/ol.2014.2471

**Published:** 2014-08-21

**Authors:** WEIHUA CHEN, KAILI MAO, ZHONGMIN LIU, ANH TUAN DINH-XUAN

**Affiliations:** 1 Department of Clinical Physiology, Medical School, Cochin Hospital, Paris Descartes University, EA 2511, Paris 75014, France; 2 Department of Urology, Tongji University, School of Medicine, Shanghai East Hospital, Shanghai 200120, P.R. China; 3 Clinical and Translational Research Center, Tongji University, School of Medicine, Shanghai East Hospital, Shanghai 200120, P.R. China

**Keywords:** RhoA, ROCK, angiogenesis, prostate cancer

## Abstract

Prostate cancer (PCa) remains a major cause of mortality among males in western countries, with little change in mortality rates observed over the past 25 years. Despite recent advances in therapy, treatment options for metastatic castration-resistant disease remain limited. In terms of chemotherapy, only the combination of docetaxel and prednisone has been shown to improve survival in these patients, but duration of response to therapy is short. There is a continuing unmet need for new systemic interventions that act either alone or synergistically with chemotherapy in patients with progressive PCa. Angiogenesis plays a critical role in tumor growth and metastasis in PCa. Several strategies have been used to target angiogenesis; however, it is becoming increasingly apparent that current anti-angiogenic therapies frequently achieve only modest effects in clinical settings. The RhoA/Rho kinase (ROCK) pathway plays a crucial role in the process of angiogenesis in PCa, and studies have demonstrated that ROCK inhibitors decrease VEGF-induced angiogenesis and tumor cell growth. However, further research is required to fully elucidate the molecular mechanisms involved in this pathway, and the potential value of modulating these mechanisms in the treatment of PCa. This study reviews the current understanding of the role of the RhoA/ROCK pathway in the process of angiogenesis in PCa, and the potential of this pathway as a therapeutic target in the future.

## 1. Introduction

Prostate cancer (PCa) is the most common malignancy and the second leading cause of cancer-related mortality in males in western countries. Advanced and metastatic stages of the disease were present in 35% of 1,589 patients with PCa diagnosed by autopsy (1). Among those patients with localized cancer who are able to receive radical prostatectomy (RP), ~35% will develop a recurrence (metastatic disease) within the 10 years following surgery (2,3). For those who present at initial diagnosis or progress with advanced or metastatic disease, androgen deprivation therapy (ADT) can be effective. However, the median duration of response to ADT is limited to between 8 months and 3 years (4), and these patients will eventually become castration resistant. Another effective treatment for castration-resistant PCa is chemotherapy, although the median duration of response is only 10.3 months (5). Therefore, there is a clear and urgent need to develop additional systemic interventions for patients with progressive PCa.

## 2. Role of angiogenesis in prostate cancer

### Angiogenic switch

Angiogenesis plays a critical role in PCa progression and metastasis. Without neovascularization, tumor growth will stop at a diameter of 2–3 mm (6). Once tumor cells are able to make their own new blood vessels, they can further expand and metastasize in a process termed the ‘angiogenic switch’ (7). The angiogenic switch in tumors is due to a shift in the balance towards neovascularization, when pro-angiogenic factors outweigh anti-angiogenic factors (8). Cancer cells and other cells in tumor tissue, such as macrophages and fibroblasts, can secrete pro-angiogenic factors, including vascular endothelial growth factor (VEGF), basic fibroblast growth factor and interleukin 8, which promote the formation of new blood vessels, causing an increase in microvessel density (MVD) in cancer tissue.

### MVD

MVD has been found to be greater in PCa than in benign prostatic hyperplasia (BPH) and normal tissue (9,10). It has been reported that MVD increases with increasing Gleason score in Pca, particularly in the poorly differentiated type (11). The MVD was also significantly correlated with cancer-specific survival in 221 patients with PCa followed for a median of 15 years (12). In other studies, mean MVD was found to correlate with increasing Gleason score and disease progression (from extraprostatic extension to metastasis) in PCa (13,14). Weidner *et al* showed that MVD was significantly higher in PCa samples from patients with metastatic disease, compared with those from patients without metastatic disease (11). Results from another study indicated that PCa angiogenesis correlated with progression after RP in patients with a Gleason score >7 (15). These data highlight the important role of angiogenesis in the progression and metastasis of PCa. MVD counts have also been shown to potentially predict tumor progression from high-grade intraepithelial neoplasia to PCa, as well as outcome in patients undergoing RP (16).

### VEGF and angiogenesis in PCa

As tumors undergo an angiogenic switch, many pro-angiogenic growth factors are secreted. Among these, VEGF is the most significant and also the most prominent regulator of physiological angiogenesis. Cells in tumor tissue, including cancer cells, fibroblasts and macrophages, secrete VEGF to stimulate new vessel formation in response to hypoxia (17,18).

Clinical studies comparing PCa with BPH revealed that VEGF expression was correlated with increased levels of angiogenesis, and that this was at least partly mediated by VEGF (10). In PCa, serum levels of the humoral ligand, VEGF, have been found to be significantly higher in patients with metastatic disease, compared with those patients with localized disease or healthy controls (19). Plasma VEGF levels have also been found to be an independent prognostic factor in males with metastatic PCa (20). Peyromaure *et al* compared 17 patients who developed bone metastases after RP with 23 patients who remained disease free and found that the expression of VEGF was significantly higher in the former group (21). Levels of VEGF in serum, plasma or urine are correlated with patient outcome in both localized as well as disseminated PCa (22). In a study of 50 patients with locally advanced disease treated with radical radiotherapy, Green *et al* reported a correlation between higher VEGF expression and worse disease-specific survival (23). In addition, levels of the VEGFR cognate receptor were found to be associated with a poorer grade of tumor differentiation and prognosis in PCa (24). Given these findings, angiogenesis inhibition has been targeted as a strategy to treat PCa. However, it has become increasingly apparent that current anti-angiogenic therapy targeting VEGF elicits only modest effects in clinical settings.

## 3. Role of the RhoA/Rho kinase pathway in angiogenesis

### RhoA and its effector Rho kinase (ROCK)

#### RhoA and its activation

RhoA is a small guanosine triphosphate hydrolase (GTPase) belonging to the Ras homology (Rho) family. The Rho family of GTPases comprise at least 23 members (25,26), which serve as key regulators of extracellular stimulus-mediated signaling networks involved in a diversity of cellular processes including motility, mitosis, proliferation and apoptosis (27). RhoA promotes actin stress fiber formation and focal adhesion assembly.

**Figure 1:**
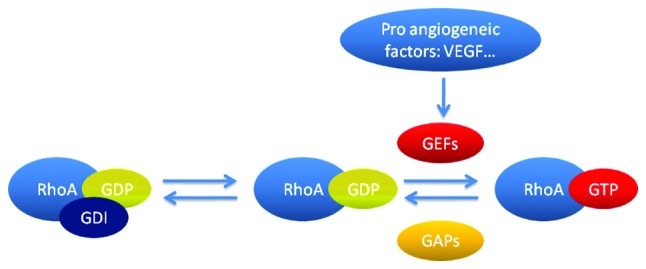
Regulation of RhoA. RhoA functions as a molecular switch, cycling between an active GTP-bound conformation and an inactive GDP-bound conformation. Intrinsic phosphatase activity hydrolyzes GTP to GDP, deactivating RhoA function, and this process is accelerated by interaction with GAPs. Conversely, interaction with GEFs facilitates the exchange of GDP to GTP, which restores the activation of RhoA. RhoA is also regulated by GDIs, which can inhibit the exchange of GDP to GTP. Pro-angiogenic factors may activate RhoA by GEFs. GTP, guanosine triphosphate; GDP, guanosine diphosphate; GAPs, GTPase-activating proteins; GEFs, guanine-nucleotide exchange factors; GDIs, guanine nucleotide dissociation inhibitors; VEGF, vascular endothelial growth factor.

Rho GTPases function as molecular switches, cycling between an active GTP-bound conformation and an inactive guanosine diphosphate (GDP)-bound conformation (28,29). When binding with GTP, they interact with downstream effectors to propagate signal transduction (30). Intrinsic phosphatase activity hydrolyzes GTP to GDP, deactivating RhoA function, and this process is accelerated by interaction with GTPase-activating proteins. Conversely, interaction with guanine-nucleotide exchange factors facilitates the exchange of GDP to GTP, which restores the activation of RhoA. The relative affinity difference of the effector molecules between the GTP- and GDP-bound states of the Rho GTPase can be as much as 100-fold, resulting in a highly-specific interaction only with the GTP-bound activated state (Fig. 1). In addition, Rho proteins are also regulated by guanine nucleotide dissociation inhibitors (GDIs), which can inhibit both the exchange of GDP to GTP and the hydrolysis of bound GTP. In the majority of cases, Rho proteins are post-translationally modified at their C-termini by prenylation of a conserved cysteine, and this facilitates their attachment to cell membranes.

#### ROCK and its function

Rho kinase (ROCK) is a serine/threonine kinase with a molecular mass of ~160 kDa, which has been shown to be the principle downstream target of RhoA. There are two ROCK isoforms: ROCK1 (ROCKβ or p160 ROCK) and ROCK2 (ROCKα or Rho kinase). ROCK1 and ROCK2 show an overall homology of 65% in their amino acid sequence and 92% in their kinase domains. Both ROCK1 and ROCK2 are expressed in vascular endothelial cells (ECs) (31,32).

When bound and activated by RhoA, ROCK translocates from the cytoplasm to the cell membrane, where it increases phosphorylation of the myosin light chain (MLC) of myosin II. This is achieved either by direct phosphorylation, or by phosphorylation of the regulatory myosin-binding subunit of myosin phosphatase (also known as the phosphatase-targeting subunit), which inhibits the phosphatase activity of this molecule (33). This, in turn, enhances actin binding and the actin-induced adenosine triphosphatase activity of myosin, facilitating the interaction of myosin with F-actin, and ultimately cell contractility. ROCK proteins can also phosphorylate cofilin indirectly via LIM kinase, and this facilitates the organization of F-actin into stress fibers and re-arrangement of the actin cytoskeleton (30).

#### RhoA/ROCK pathway and cell motility

In eukaryotes, organization and reorganization of the cytoskeleton underpins cellular morphology and motility. The actin cytoskeleton is composed of actin filaments and numerous specialized actin-binding proteins. Actin filaments are created by the simple polymerization of actin monomers, regulated dynamically by numerous upstream signaling pathways, notably Rho GTPases (30). A coordinated regulation of the actin network is essential to produce directed cell movement.

During cell movement, RhoA is active at the trailing edge of the cell to promote retraction, while Rac, another member of the Rho family, is active at the leading edge, promoting protrusion. Active RhoA has also been shown to localize at the leading edge of migrating cells (34–36), indicating that RhoA not only acts in retraction, but also regulates protrusion at the front of the cell. Notably, increases in RhoA activity were found to be correlated with increases in protrusion rates, and were synchronous with cell-edge advancement (36). These data highlight the important role of RhoA in cell movement.

### Association between the RhoA/ROCK pathway and angiogenesis

#### Mechanism of angiogenesis

**Figure 2:**
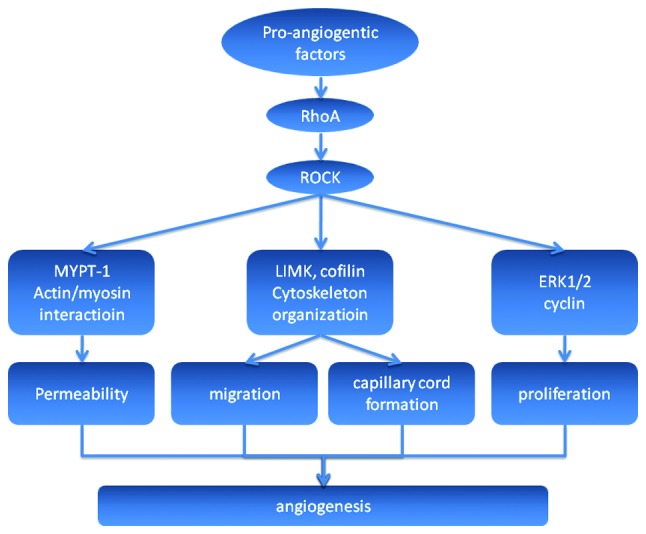
Illustration of the RhoA/ROCK pathway in angiogenesis. Pro-angiogenic factors can activate the RhoA/ROCK pathway then subsequently activate the downstream molecules that take part in the multisteps of angiogenesis. Firstly, MYPT-1 can be activated by ROCK and cause myosin light chain phosphorylation followed by actin-mediated EC contraction, which leads to an increase in the permeability of the basement membrane allowing a new capillary to sprout. Other molecules involved in cytoskeletal organization, such as LIMK and cofilin, are activated and cause ECs to migrate into the extracellular matrix towards the angiogenic stimulus. The leading front of migrating cells is driven by enhanced proliferation of ECs, in which ERK1/2 and cyclin may play a role when activated by ROCK. This is followed by re-organization of ECs to form tubules with a central lumen, which finally reorganize to result in new capillary stabilization. ROCK, Rho kinase; MYPT-1, myosin phosphatase target subunit 1; ECs, endothelial cells; ERK1/2, extracellular signal-regulated kinase 1/2.

Angiogenesis is a five-step process involving a complex series of events. Firstly, an increase in the permeability of the basement membrane allows a new capillary to sprout. Next, ECs activated by angiogenic factors migrate through the basement membrane into the extracellular matrix, towards the angiogenic stimulus. The leading front of migrating cells is driven by enhanced proliferation of ECs. This is then followed by re-organization of ECs to form tubules with a central lumen, together with the recruitment of periendothelial cells (pericytes) and vascular smooth muscle cells for new capillary stabilization (37). The RhoA/ROCK pathway plays a role in each of these key steps (Fig. 2).

#### Permeability

The endothelium is a semi-permeable barrier that lines the vasculature, comprising ECs that are connected to each other by interendothelial junctions, consisting of protein complexes organized as tight junctions and adherent junctions. The latter are in the majority (38), and are composed of vascular endothelial (VE) cadherin that associates homotypically with VE-cadherin on adjacent cells. VE-cadherin binds to the actin cytoskeleton. Actin-mediated EC contraction occurs as a result of MLC phosphorylation, and this can cause dysfunction of the endothelial barrier by inducing the formation of small gaps between neighboring cells (39). RhoA, through its downstream effector ROCK, plays a role in endothelial barrier dysfunction by potentiating MLC phosphorylation via inhibition of MLC phosphatase activity. Studies have also confirmed that RhoA contributes to VEGF-induced hyperpermeability in the endothelium (40).

#### Migration

The formation of stress fibers and cellular contraction is essential for EC migration, and these processes are mediated by Rho GTPases (41). van Nieuw Amerongen *et al* demonstrated *in vitro* that VEGF induces the activation of RhoA and this increase in RhoA activity is necessary for VEGF-induced reorganization of the F-actin cytoskeleton. This process can be inhibited by transfection of ECs with a RhoA dominant-negative mutant vector or by a RhoA inhibitor C3 (42). Zhao *et al* showed that increased expression of RhoA in human umbilical vein ECs significantly enhanced cytoskeletal reorganization of transfected cells, cell migration and angiogenic capacity, which suggests that RhoA plays a key part in these processes *in vitro* (43).

#### Proliferation

Several lines of evidence suggest that Rho proteins play an important role in normal and cancerous cell growth processes, including G1 phase cell cycle progression and mitogenesis (44). Cytokinesis is a step in mitogenesis which is critical within the cell cycle. In eukaryotic cells, cytokinesis requires an actin and myosin contractile ring, which constricts and cleaves the cell, forming two daughter cells. Inhibition of Rho GTPases prevents the assembly of this contractile ring in a variety of mammalian cells. Expression of constitutively activated Rho GTPases also blocks cytokinesis, suggesting that cycling between the active and inactive forms is required for its function (45).

The role of RhoA signaling in cell survival has been evaluated in several non-EC cell types. Results showed that inhibition of Rho signaling leads to apoptosis via alterations in cell adhesion and the induction of p53 and other pro-apoptotic proteins, or via ceramide upregulation leading to caspase cleavage and subsequent activation (46,47). Studies have shown that the ROCK inhibitors, fasudil and Y-27632, not only inhibit VEGF-induced cell proliferation, but also reverse the protective effect of VEGF on apoptosis, which results in a decrease in viability of VEGF-stimulated ECs (48,49). Data obtained with these inhibitors have revealed the important role of the RhoA/ROCK pathway in EC proliferation and cell viability.

#### Morphogenesis

Cultured ECs can undergo marked changes in shape and tube formation that closely imitate pre-capillary cord formation *in vivo* (50). *In vitro* angiogenesis assays found that the mean tube length of the capillary-like tubular structures formed by ECs was reduced by transfection of a RhoA dominant-negative mutant vector, the RhoA inhibitor C3, or the ROCK inhibitor Y-27632 (42). In another study, Zhao *et al* demonstrated that overexpression of RhoA increased the tube length in transfected ECs (43).

## 4. RhoA/Rho kinase pathway and angiogenesis in prostate cancer

As discussed previously, the RhoA/ROCK pathway participates in the process of angiogenesis in numerous types of cancer, including PCa. Tumor blood vessels always exhibit abnormal structure and function. A study investigating the ECs of mice carrying the transgenic adenocarcinoma of the mouse prostate transgene revealed that the aberrant mechanosensing of extracellular matrix cues and resulting abnormal responses in these cells correlated with a constitutively high level of baseline activity of Rho GTPase and its downstream effector, ROCK (51). These findings highlighted the important role of the RhoA/ROCK pathway in the angiogenesis of PCa. A number of other studies have also demonstrated that RhoA/ROCK pathway inhibitors decrease angiogenesis and cell growth in PCa (52–54).

In an *in vitro* study, Y-27632 inhibited metastatic growth of highly invasive PC3 cells in immunocompromised mice (52). Another ROCK inhibitor, Wf-536, greatly enhanced the *in vitro* inhibition of EC migration, vacuolation, lumen and cord formation, and VEGF- and hepatocyte growth factor-stimulated endothelial sprout formation, when combined with the matrix metalloproteinase inhibitor, marimastat (53). Early treatment with a combination of Wf-536 plus marimastat, with or without paclitaxel, of immunocompromised mice bearing xenotransplants of PC3 cells was associated with significant inhibition of tumor growth and increased tumor necrosis (53). Certain potential anti-angiogenic medications, such as anacardic acid, have been found to inhibit human prostate tumor xenograft angiogenesis by targeting the Rho GTPase signaling pathway (54).

## 5. Implications for therapy

VEGF antibody bevacizumab has been approved for numerous cancer therapies, such as for colon, lung and kidney cancer. One of its main side effects is hypertension, while ROCK inhibitors have vasodilation effects. For example, fasudil has shown favorable effects in patients with angina (55) and pulmonary hypertension (56) in clinical trials and has been approved for cerebral vasospasm by the inhibition of vessel contraction. Their effects on anti-angiogenesis have also gradually been discovered, as we have reviewed above. It may be feasible to combine the ROCK inhibitor with VEGF antibody in order to enforce their anti-angiogenesis effects while reducing the side effects.

## 6. Conclusion

Inhibition of angiogenesis has been targeted as a strategy to treat PCa. Until now, the main focus has been on the VEGF pathway. However, it has become increasingly apparent that current anti-angiogenic therapy elicits only modest effects in clinical settings. The RhoA/ROCK pathway plays a crucial role in cancer angiogenesis and should also be a potential target for anti-angiogenic therapy. Additional studies are required to elucidate the molecular mechanisms of the RhoA/ROCK pathway in PCa angiogenesis, and the potential value of modulating these mechanisms for the treatment of PCa.

## References

[1] Bubendorf L, Schöpfer A, Wagner U (2000). Metastatic patterns of prostate cancer: an autopsy study of 1,589 patients. Hum Pathol.

[2] Roehl KA, Han M, Ramos CG, Antenor JA, Catalona WJ (2004). Cancer progression and survival rates following anatomical radical retropubic prostatectomy in 3,478 consecutive patients: long-term results. J Urol.

[3] Hull GW, Rabbani F, Abbas F, Wheeler TM, Kattan MW, Scardino PT (2002). Cancer control with radical prostatectomy alone in 1,000 consecutive patients. J Urol.

[4] Daneshgari F, Crawford ED (1993). Endocrine therapy of advanced carcinoma of the prostate. Cancer.

[5] Eymard JC, Oudard S, Gravis G (2010). Docetaxel reintroduction in patients with metastatic castration-resistant docetaxel-sensitive prostate cancer: a retrospective multicentre study. BJU Int.

[6] Folkman J, Cole P, Zimmerman S (1966). Tumor behavior in isolated perfused organs: in vitro growth and metastases of biopsy material in rabbit thyroid and canine intestinal segment. Ann Surg.

[7] Banerjee S, Dowsett M, Ashworth A, Martin LA (2007). Mechanisms of disease: angiogenesis and the management of breast cancer. Nat Clin Pract Oncol.

[8] Bergers G, Benjamin LE (2003). Tumorigenesis and the angiogenic switch. Nat Rev Cancer.

[9] Bigler SA, Deering RE, Brawer MK (1993). Comparison of microscopic vascularity in benign and malignant prostate tissue. Hum Pathol.

[10] Stefanou D, Batistatou A, Kamina S, Arkoumani E, Papachristou DJ, Agnantis NJ (2004). Expression of vascular endothelial growth factor (VEGF) and association with microvessel density in benign prostatic hyperplasia and prostate cancer. In Vivo.

[11] Weidner N, Carroll PR, Flax J, Blumenfeld W, Folkman J (1993). Tumor angiogenesis correlates with metastasis in invasive prostate carcinoma. Am J Pathol.

[12] Borre M, Offersen BV, Nerstrom B, Overgaard J (1998). Microvessel density predicts survival in prostate cancer patients subjected to watchful waiting. Br J Cancer.

[13] Brawer MK, Deering RE, Brown M, Preston SD, Bigler SA (1994). Predictors of pathologic stage in prostatic carcinoma. The role of neovascularity. Cancer.

[14] Bostwick DG, Wheeler TM, Blute M (1996). Optimized microvessel density analysis improves prediction of cancer stage from prostate needle biopsies. Urology.

[15] Silberman MA, Partin AW, Veltri RW, Epstein JI (1997). Tumor angiogenesis correlates with progression after radical prostatectomy but not with pathologic stage in Gleason sum 5 to 7 adenocarcinoma of the prostate. Cancer.

[16] Bono AV, Celato N, Cova V, Salvadore M, Chinetti S, Novario R (2002). Microvessel density in prostate carcinoma. Prostate Cancer Prostatic Dis.

[17] Byrne AM, Bouchier-Hayes DJ, Harmey JH (2005). Angiogenic and cell survival functions of vascular endothelial growth factor (VEGF). J Cell Mol Med.

[18] Dvorak HF, Detmar M, Claffey KP, Nagy JA, van de Water L, Senger DR (1995). Vascular permeability factor/vascular endothelial growth factor: an important mediator of angiogenesis in malignancy and inflammation. Int Arch Allergy Immunol.

[19] Duque JL, Loughlin KR, Adam RM, Kantoff PW, Zurakowski D, Freeman MR (1999). Plasma levels of vascular endothelial growth factor are increased in patients with metastatic prostate cancer. Urology.

[20] George DJ, Halabi S, Shepard TF (2001). Cancer and Leukemia Group B 9480: Prognostic significance of plasma vascular endothelial growth factor levels in patients with hormone-refractory prostate cancer treated on Cancer and Leukemia Group B 9480. Clin Cancer Res.

[21] Peyromaure M, Camparo P, Badoual C, Descazeaud A, Dinh-Xuan AT (2007). The expression of vascular endothelial growth factor is associated with the risk of cancer progression after radical prostatectomy. BJU Int.

[22] Bok RA, Halabi S, Fei DT (2001). Vascular endothelial growth factor and basic fibroblast growth factor urine levels as predictors of outcome in hormone-refractory prostate cancer patients: a cancer and leukemia group B study. Cancer research.

[23] Green MM, Hiley CT, Shanks JH (2007). Expression of vascular endothelial growth factor (VEGF) in locally invasive prostate cancer is prognostic for radiotherapy outcome. Int J Radiat Oncol Biol Phys.

[24] Huss WJ, Hanrahan CF, Barrios RJ, Simons JW, Greenberg NM (2001). Angiogenesis and prostate cancer: identification of a molecular progression switch. Cancer Res.

[25] Wennerberg K, Rossman KL, Der CJ (2005). The Ras superfamily at a glance. J Cell Sci.

[26] Bustelo XR, Sauzeau V, Berenjeno IM (2007). GTP-binding proteins of the Rho/Rac family: regulation, effectors and functions in vivo. Bioessays.

[27] Etienne-Manneville S, Hall A (2002). Rho GTPases in cell biology. Nature.

[28] Bourne HR, Sanders DA, McCormick F (1991). The GTPase superfamily: conserved structure and molecular mechanism. Nature.

[29] Bourne HR, Sanders DA, McCormick F (1990). The GTPase superfamily: a conserved switch for diverse cell functions. Nature.

[30] Spiering D, Hodgson L (2011). Dynamics of the Rho-family small GTPases in actin regulation and motility. Cell Adh Migr.

[31] Horowitz S, Binion DG, Nelson VM (2007). Increased arginase activity and endothelial dysfunction in human inflammatory bowel disease. Am J Physiol Gastrointest Liver Physiol.

[32] Ming XF, Barandier C, Viswambharan H (2004). Thrombin stimulates human endothelial arginase enzymatic activity via RhoA/ROCK pathway: implications for atherosclerotic endothelial dysfunction. Circulation.

[33] Somlyo AP, Somlyo AV (2003). Ca2^+^ sensitivity of smooth muscle and nonmuscle myosin II: modulated by G proteins, kinases and myosin phosphatase. Physiological reviews.

[34] Pertz O, Hodgson L, Klemke RL, Hahn KM (2006). Spatiotemporal dynamics of RhoA activity in migrating cells. Nature.

[35] Kurokawa K, Matsuda M (2005). Localized RhoA activation as a requirement for the induction of membrane ruffling. Mol Biol Cell.

[36] Machacek M, Hodgson L, Welch C (2009). Coordination of Rho GTPase activities during cell protrusion. Nature.

[37] Sakamoto S, Ryan AJ, Kyprianou N (2008). Targeting vasculature in urologic tumors: mechanistic and therapeutic significance. J Cell Biochem.

[38] Mehta D, Malik AB (2006). Signaling mechanisms regulating endothelial permeability. Physiol Rev.

[39] Dudek SM, Garcia JG (2001). Cytoskeletal regulation of pulmonary vascular permeability. J Appl Physiol (1985).

[40] Sun H, Breslin JW, Zhu J, Yuan SY, Wu MH (2006). Rho and ROCK signaling in VEGF-induced microvascular endothelial hyperpermeability. Microcirculation.

[41] Kiosses WB, Daniels RH, Otey C, Bokoch GM, Schwartz MA (1999). A role for p21-activated kinase in endothelial cell migration. J Cell Biol.

[42] van Nieuw Amerongen GP, Koolwijk P, Versteilen A, van Hinsbergh VW (2002). Involvement of RhoA/Rho kinase signaling in VEGF-induced endothelial cell migration and angiogenesis in vitro. Aterioscler Thromb Vasc Biol.

[43] Zhao L, Xu G, Zhou J (2006). The effect of RhoA on human umbilical vein endothelial cell migration and angiogenesis in vitro. Oncol Rep.

[44] Van Aelst L, D’Souza-Schorey C (1997). Rho GTPases and signaling networks. Genes Dev.

[45] Glotzer M (2001). Animal cell cytokinesis. Annu Rev Cell Dev Biol.

[46] Bobak D, Moorman J, Guanzon A, Gilmer L, Hahn C (1997). Inactivation of the small GTPase Rho disrupts cellular attachment and induces adhesion-dependent and adhesion-independent apoptosis. Oncogene.

[47] Petrache I, Crow MT, Neuss M, Garcia JG (2003). Central involvement of Rho family GTPases in TNF-alpha-mediated bovine pulmonary endothelial cell apoptosis. Biochem Biophys Res Commun.

[48] Yin L, Morishige K, Takahashi T (2007). Fasudil inhibits vascular endothelial growth factor-induced angiogenesis in vitro and in vivo. Mol Cancer Ther.

[49] Bryan BA, Dennstedt E, Mitchell DC (2010). RhoA/ROCK signaling is essential for multiple aspects of VEGF-mediated angiogenesis. FASEB J.

[50] Montesano R, Orci L, Vassalli P (1983). In vitro rapid organization of endothelial cells into capillary-like networks is promoted by collagen matrices. J Cell Biol.

[51] Ghosh K, Thodeti CK, Dudley AC, Mammoto A, Klagsbrun M, Ingber DE (2008). Tumor-derived endothelial cells exhibit aberrant Rho-mediated mechanosensing and abnormal angiogenesis in vitro. Proc Natl Acad Sci USA.

[52] Somlyo AV, Bradshaw D, Ramos S, Murphy C, Myers CE, Somlyo AP (2000). Rho-kinase inhibitor retards migration and in vivo dissemination of human prostate cancer cells. Biochem Biophys Res Commun.

[53] Somlyo AV, Phelps C, Dipierro C (2003). Rho kinase and matrix metalloproteinase inhibitors cooperate to inhibit angiogenesis and growth of human prostate cancer xenotransplants. FASEB J.

[54] Wu Y, He L, Zhang L (2011). Anacardic acid (6-pentadecylsalicylic acid) inhibits tumor angiogenesis by targeting Src/FAK/Rho GTPases signaling pathway. J Pharmacol Exp Ther.

[55] Shimokawa H, Hiramori K, Iinuma H (2002). Anti-anginal effect of fasudil, a Rho-kinase inhibitor, in patients with stable effort angina: a multicenter study. J Cardiovasc Pharmacol.

[56] Fukumoto Y, Matoba T, Ito A (2005). Acute vasodilator effects of a Rho-kinase inhibitor, fasudil, in patients with severe pulmonary hypertension. Heart.

